# Sociodemographic Factors Associated with Autistic Youth’s Psychotherapy Service Use

**DOI:** 10.1007/s10488-025-01448-9

**Published:** 2025-06-03

**Authors:** Jessica V. Smith, Rose Nevill, Pamela B. DeGuzman, Michelle Menezes, Micah O. Mazurek

**Affiliations:** 1 Department of Human Services School of Education and Human Development, University of Virginia, VA, USA; 2 School of Nursing, University of Virginia, VA, USA; 3 Child Study Center, Yale School of Medicine, CT, USA

**Keywords:** Autism, Psychotherapy services, Service access, Claims data, Mental health, Social determinants of health

## Abstract

Autistic youth have a high co-occurrence of mental health challenges and a resultant high need for mental health treatment. However, they experience mental health service disparities compared to non-autistic youth. Social determinants of health (SDH) may contribute to mental health service disparities among autistic youth, yet this has not been previously examined. Therefore, the present study utilized a validated composite of children’s SDH to examine whether autistic youth with better SDH were more likely to use psychotherapy services compared to those with poor SDH using state-level records of insurance billing claims data (2019 All-Payer Claims Database). 700 autistic youth with a co-occurring mental health condition were included in analyses. One or more claim records for psychotherapy CPT codes were used as indicators of psychotherapy service use. SDH was assessed using the Childhood Opportunity Index, a continuous composite measure of neighborhood SDH. The predictive effect of SDH on psychotherapy use were examined, while examining covariates of insurance type and age. Approximately 70% of the sample did not use any psychotherapy services. Inconsistent with findings from non-autistic samples, autistic youth’s SDH did not predict their likelihood of using psychotherapy services; rather, those with low and high opportunity alike did not access psychotherapy. Age increased the likelihood, and using Medicaid decreased the likelihood of psychotherapy service use. These results may highlight the compounded barriers to psychotherapy that autistic youth may experience, including the paucity of mental health providers who accept Medicaid and accept autistic youth clients.

**Supplementary Information:**

The online version contains supplementary material available at 10.1007/s10488-025-01448-9.

Autism spectrum disorder (hereby referred to as “autism”) is a neurodevelopmental condition affecting approximately one in 36 children in the United States (US), with hallmark diagnostic features including challenges with social communication and interaction and the presence of restricted and repetitive patterns of behavior, interests, or activities (American Psychiatric Association, 2013; Maenner et al., 2023). The autism spectrum is heterogeneous, encompassing individuals with varying levels of intellectual functioning, adaptive skills, and communication styles. This heterogeneity also contributes to the wide variation in priorities and clinical needs among those with autism, including autistic youth (< 18 years of age) who are the focus of the present study. For example, due to their wide array of needs, many autistic youth engage in a range of therapies, such as physical therapy (PT), occupational therapy (OT), applied behavioral analysis (ABA), and/or speech therapy (Thomas et al., 2007).

## Autism & Mental Health Needs

In addition to core diagnostic features of autism, 95% of autistic youth^1^ have a co-occurring mental health condition (Bougeard et al., 2021). Rates of mental health conditions in children and adolescents are higher in autistic samples than in the general population (Bougeard et al., 2021). For example, estimates from a population-based study of eight-year-old autistic children indicated that 75% had co-occurring mood disorder, 32% oppositional defiant disorder, 26% co-occurring attention deficit-hyperactivity disorder (ADHD), and 10% co-occurring anxiety (Soke et al., 2018). A recent systematic review of the prevalence of selected co-occurring conditions in autistic youth up to age 18 years identified rates as high as 86% for co-occurring ADHD and 82% for an anxiety disorder (Bougeard et al., 2021). The prevalence of co-occurring mental health conditions was even higher in a study of youth receiving publicly funded or school-based mental health services in California (Brookman-Frazee et al., 2018).

Given these high rates of co-occurring conditions, autistic youth have a significant need for effective mental health treatment. In the general population, the non-pharmacological mental health interventions with the most evidence to date are psychotherapy-based, including cognitive behavioral therapy for internalizing disorders such as anxiety or depression (American Psychological Association, 2019; Bandelow et al., 2017) or parent behavior management training for externalizing symptoms such as disruptive behaviors (Kaminski & Claussen, 2017). The evidence base for mental health interventions in autistic youth, though much smaller, mirrors that of the general population (Dickson et al., 2021; see for review: Menezes et al., 2020; Vasa et al., 2014).

## Mental Health Service Access for Autistic Youth

Despite their high mental health needs and the demonstrated effectiveness of mental health interventions to meet those needs (e.g., Wood et al., 2020), youth on the spectrum experience challenges in accessing mental health services. On the surface, some study findings highlight high rates of mental health service use (Chiri & Warfield, 2012; Narendorf et al., 2011). For instance, youth on the spectrum had a significantly higher volume of mental health service use claims (e.g., medication management, case management, evaluation and assessment) and duration of services overall compared to a sample of non-autistic peers matched on demographic and treatment setting characteristics. However, when focusing on specific treatment services, autistic youth had fewer psychotherapy claims than their non-autistic peers (Stadnick et al., 2020). These disparities may, in part, be attributable to the high rate of medication management for autistic youth with a co-occurring mental health condition (Jobski et al., 2017; Rast et al., 2023). In addition, autistic youth often receive multiple therapies for co-occurring conditions or delays (e.g., OT, PT, speech therapy), which may leave little time for additional appointments with mental health clinicians. However, further research is needed to fully understand these low rates of psychotherapy, given that psychotherapy is the best practice treatment for most mental health conditions.

Born out of the many conceptualizations of service access in the public health literature, Levesque and colleagues (2013) defined service access as “the opportunity to have health needs fulfilled (p. 4).” The opportunity to have mental health needs fulfilled is crucial for promoting long-term outcomes, yet autistic youth with co-occurring mental health conditions have higher rates of unmet service needs compared to those without a co-occurring condition (Zablotsky et al., 2015). Researchers have coined this phenomenon as the “autism mental health crisis,” highlighting a juxtaposition between the high rates of co-occurring mental health conditions and the “low chance of receiving effective help” (Mandy, 2022, p. 289). Few mental health facilities and providers deliver mental health services to autistic clients (Cantor et al., 2020, 2022; Cervantes et al., 2023; Maddox et al., 2021), as providers often lack training in effective delivery of mental health services for autistic clients (Cervantes et al., 2023). Some of the caregiver- and self-reported barriers to accessing mental health treatment services include lengthy waitlists, service costs, professionals’ lack of autism knowledge, unwillingness to make autism-specific adaptations, lack of recognition or belief in the autistic individuals’ mental health challenges, and being “bounced” between services (Adams & Young, 2021).

Individual sociodemographic factors may also impact mental health and mental health care access. For example, autistic youth living in poverty have poorer mental health (Flouri et al., 2015; Midouhas et al., 2013) and greater difficulties accessing services broadly (Smith et al., 2020; Thomas et al., 2007), due in part to lower knowledge of available services and logistical barriers to care (e.g., lack of transportation, inability to take time off for appointments). However, one study contradicts these findings and suggests that autistic youth with anxiety who had lower socioeconomic status had fewer problems accessing mental health services compared to those with greater financial means (Conrad et al., 2022), perhaps due to their ability to access needs-based programs.

Autistic youth with minoritized ethno-racial identities also have greater difficulty accessing mental health services (Bilaver et al., 2021; Magaña et al., 2013), due to factors such as the limited number of culturally or linguistically matched providers and cultural differences in beliefs about mental health and mental health treatment. Age also relates to mental health service use, given that youth are also more likely to use mental health services as they age and adolescent-onset conditions emerge (Lai et al., 2019; Ryan et al., 2018; Song et al., 2022; Turcotte et al., 2016). Finally, those living in a nonmetropolitan area also have greater difficulty accessing mental health services, including psychotherapeutic treatment services (Drahota et al., 2020; Thomas et al., 2007), because of the far distance to available mental health providers and the consequential need for time and transportation resources. Notably, these barriers are consistent with those reported in non-autistic samples (e.g., Anderson et al., 2017; Gulliver et al., 2010; Reardon et al., 2017).

### Social Determinants of Health

The aforementioned “nonmedical factors” that impact health outcomes have been conceptualized as social determinants of health (SDH; CDC, 2024). The World Health Organization (WHO) commission on SDH defines SDH as the “circumstances in which people are born, grow, live, work, and age” (Solar, 2010). Health policy researchers and agencies like the WHO and the Centers for Disease Control have urged researchers to incorporate SDH into research addressing health disparities, as researchers suggest that SDH are a mechanism underlying the relationship between one’s socio-political context (e.g., policies, programs) and individual-level health outcomes (CDC, 2024; Solar, 2010). Five domains comprise SDH including education access and quality (e.g., literacy and graduation rates), health care access and quality (e.g., access to medical facilities, health literacy), neighborhood and built environment (e.g., access to food, quality of housing, crime), social and community context (e.g., social cohesion, incarceration, discrimination), and economic stability (e.g., employment, poverty; HHS, n.d.).

There has been a recent call to action for autism researchers to focus on SDH to better understand autism-specific health disparities and barriers to services (Hotez & Shea, 2023). Prior studies of SDH have been limited in their ability to evaluate SDH’s impact on service use because of fragmentary characterization of SDH (i.e., focusing on a few aspects of or related to SDH). For instance, studies evaluating SDH in autistic youth have ranged from using combinations of ethno-racial identity, household income, parental education level, or socioeconomic status (Smith et al., 2020; Zuckerman et al., 2015) to administering caregiver-report SDH screeners (Jafarabadi et al., 2021). Importantly, each of these studies have focused on one dimension of or related to SDH, such as marginalized identities (e.g., sex, race/ethnicity) or individual family’s reports of food or housing insecurity.

Studies using multidimensional measures of SDH are needed to fully understand barriers to mental healthcare among autistic youth, and to aid in the development of policy and capacity-building initiatives. The Child Opportunity Index (COI; Acevedo-Garcia et al., 2014) holds particular promise as a validated measure of SDH that has been used in recent public health research (e.g., Bettenhausen et al., 2022; Fritz et al., 2022). The COI is a multidimensional index of “neighborhood-based conditions and resources conducive to healthy child development” (Acevedo-Garcia et al., 2014, p. 1948). Unlike other measures of area SDH, such as the Area Deprivation Index (Singh, 2003), the COI was particularly designed for use with children (Zolotor et al., 2023). Specific dimensions of the COI include educational opportunities (e.g., school poverty rate, proximity to high-quality early childhood education centers, student proficiency levels), health and environmental factors (e.g., proximity to healthcare facilities, toxic waste release sites, and parks and open spaces; retail healthy food environment index), and social and economic opportunities (e.g., foreclosure rate, poverty rate, proximity to employment). To date, no autism-related studies of service disparities have used the COI to evaluate SDH.

## Present Study

Despite a high co-occurrence of mental health conditions and consequential high need for treatment, autistic youth experience barriers to accessing psychotherapy services. Previous research findings highlight predictors of service disparities at each level of care (systems-, provider-, and individual-level); however, to date, research at the individual-level has been limited by piecemeal approaches to understanding sociodemographic predictors. Therefore, the present study will expand the literature on psychotherapy service use patterns of autistic youth (< 18 years of age) by conducting the first evaluation of the impact of SDH on psychotherapy service use with a multidimensional, validated measure of neighborhood SDH. We hypothesize that, among autistic youth who have mental health service needs (i.e., a co-occurring mental health condition), better SDH (i.e., higher COI scores) will predict the likelihood of psychotherapy service use, when controlling for insurance type and age.

## Methods

The present study used the 2019 Virginia All-Payer Claims Database (APCD), which is an insurance billing claims dataset governed by the Virginia Department of Health. The dataset contains paid insurance claims records for approximately four million Virginia residents with commercial, Medicaid, and Medicare coverage across all types of healthcare services (e.g., medical, psychological, allied health; Virginia Health Information, 2021). Insurance billing claims data provide opportunities to systematically understand service utilization, as it documents all services billed through insurance. The APCD is one such dataset that has been used in investigations of services related to autism (e.g., DeGuzman et al., 2022; Saloner & Barry, 2019). Due to the nature of this secondary data analysis of deidentified data, this study was determined to be exempt from review by the Institutional Review Board at the University of Virginia, and the collection of informed consent was not applicable.

## Participants

Youth (< 18 years of age) with autism and a co-occurring mental health condition were included in the present sample, which was determined by the presence of at least one claim with an accompanying International Classification of Disease, Tenth Revision (ICD-10) code of autism (F84 and appropriate subcodes) and at least one claim with an “F” code for a mental health condition for which psychotherapy is an evidence-based practice (see Table 1 for the complete list). Claims for inpatient or residential treatment were excluded from the present study, as questions related to acute care were outside of the scope of the present research question, given the need for psychotherapy in these settings is exacerbated by a sudden event or acuity in symptoms.

**Table 1:** Sample characteristics (*N* = 700)

Co-occurring mental health conditions present in claims^a^	*N* (%)	M*M* (SD)
Schizophrenia (F20.x)	2 (0.29%)	
Schizoaffective disorder (F25.x)	1 (0.14%)	
Bipolar disorder (F31.x)	31 (4.43%)	
Depressive episode (F32.x)	40 (5.71%)	
Major depressive disorder (F33.x)	17 (2.43%)	
Persistent mood disorder (F34.x)	45 (6.43%)	
Specific phobias (F40.x)	7 (1%)	
Other anxiety disorders (F41.x)	165 (23.57%)	
Obsessive-compulsive disorders (F42.x)	16 (2.29%)	
Reactive/adjustment disorders (F43.x)	58 (8.29%)	
Somatoform disorders (F45.x)	4 (0.57%)	
Eating disorders (F50.x)	6 (0.86%)	
Sexual dysfunction (F52.x)	1 (0.14%)	
Impulse disorders (F63.x)	9 (1.29%)	
Gender identity (F64.x)	3 (0.43%)	
Attention-deficit/hyperactivity disorder (F90.x)	509 (72.71%)	
Conduct disorder (F91.x)	76 (10.86%)	
Emotional disorders with onset in childhood (F93.x)	9 (1.29%)	
Disorders of social functioning (F94.x)	11 (1.57%)	
Tic disorders (F95.x)	10 (1.43%)	
Psychotherapy services use		
Did not receive psychotherapy services	490 (70%)	
Received psychotherapy services	210 (30%)	
Race		
American Indian/Alaska Native	3 (0.43%)	
Asian	13 (1.86%)	
Black/African American	131 (18.71%)	
Native Hawaiian/Other Pacific Islander	4 (0.57%)	
White	336 (48%)	
Other	196 (28%)	
Unknown/Missing	17 (2.43%)	
Ethnicity		
Hispanic/Latinx	1 (0.14%)	
Not Hispanic/Latinx	170 (24.29%)	
Unknown	529 (75.57%)	
Sex/gender^b^		
Male	550 (78.57%)	
Female	150 (21.43%)	
Patient Age		11.06 (3.62) Range: 1–17

^a^Data were not reported for mental health conditions that were not reported in any records. Participants were reported to have multiple mental health conditions, resulting in percentages exceeding 100%. ^b^ Sex/gender are used interchangeably here to denote the lack of specificity within claim records as to whether data related to patients’ sex assigned at birth or gender identity

The prevalence of the present sample’s co-occurring conditions varied from 0.14% (schizoaffective disorder, sexual dysfunction) to 72.71% (attention-deficit/hyperactivity disorder). Related to intellectual or developmental disability, 4% of the sample had co-occurring intellectual disability, 4% had co-occurring global developmental delay, and 8% had a co-occurring language disorder. The majority of the sample were male (78.57%), and 48% of the sample were White. Their average age was preadolescent (*M* = 11.06, SD = 3.62). See Table 1 for full sample characteristics.

## Measures

### Provider Characteristics

Providers of psychotherapy services were characterized by the variables available, namely the type of provider from whom the services are being claimed (e.g., psychologist, psychiatrist), what psychotherapy services they provided (e.g., individual, group), and in what setting those services were delivered (e.g., community mental health center, telehealth).

### Service Type & Use

Service type was extracted from the APCD using the Current Procedural Terminology (CPT) code corresponding with the claim. All claims were coded for the current analysis based on whether they were for a psychotherapy-based claim (see Table 2 for full list of codes and descriptions).

**Table 2:** Current procedural terminology (CPT) codes for psychotherapy services

Description of services	CPT Code
One-to-one provision of psychotherapy for the treatment of psychiatric disorders/conditions	90832, 90833, 90834, 90836, 90837, 90838
One-to-one provision of psychoanalytic therapy techniques	90845
Provision of family counseling or family therapy (both with and without patient present, one-to-one and group settings)	90846, 90847, 90849
Provision of psychotherapy within a group setting with patients who are not members of the same family	90853

At the individual level, a binary variable of “service use” was created to indicate the use of psychotherapy services if one or more of the aforementioned CPT codes were billed.

### Patient Characteristics

The nature of claims data limits the availability of demographic information about the individual in receipt of services for which insurance is being billed (hereafter, the “patient”). Consequently, patients were characterized by the variables available, namely sex, age, insurance type (Medicaid or commercial), and the co-occurring mental health conditions included in the patients’ claims. Data regarding race and ethnicity are reported, as available; however, these data lack precision in reporting (~ 30% of patients with “other” race, 75% of patients missing ethnicity data). Demographic variables (e.g., zip code, sex, age) from the individual’s *first* claim were uniformly used for all patients for consistency.

### Childhood Opportunity Index 2.0

The COI is a publicly available measure of child SDH for a given neighborhood, based on zip code (diversitydatakids.org, 2023; Noelke et al., 2020). The COI 2.0 has 29 indicators of childhood opportunity, and each indicator has individual, varying weights dependent on how strongly they predict child health outcomes. The three overall domains of the COI are education, health/environment, and social/economic. Higher scores indicate more opportunity for positive outcomes. The COI shows strong predictive validity of health outcomes, like life expectancy (Noelke et al., 2020). The present study utilized the nationally normed overall COI 2.0 score. See Supplemental Materials A for individual domains, indicators, and descriptions of the COI.

## Data Analysis Plan

All data were analyzed using R and Rstudio (Posit team, 2023; R Core Team, 2022), aided by the following packages: api2 lm, fastDummies, janitor, and tidyverse (Firke, 2024; French, 2023; Wickham et al., 2019). The present data were converted from a claims-level data set to be aggregated at the individual-level using the data associated with each accompanying patient identifier in the APCD.

To address the primary hypothesis, a hierarchical binary logistic regression was used to evaluate the impact of SDH on the likelihood of using psychotherapy services (Field et al., 2012). The first model predicted the binary indicator of service use (i.e., the presence or absence of psychotherapy billing claims) by covariates of child age and insurance type (reference group: “commercial”). The second model included the aforementioned variables and overall COI score. Model comparisons were conducted using Pearson’s *χ*^2^ goodness of fit test.

## Results

Covariates of insurance type, and age were included in the first model, resulting in significant predictive effects, *X*^2^ (2) = 43.68, *p* <.001. The second model, which added SDH as a predictor and included insurance type and age as covariates, was also significant, *X*^2^ (3) = 44.01, *p* <.001, though SDH was not a significant predictor of psychotherapy service use, *B* = 0.002, *z* = 0.57, *p* =.57. The difference between the two models was not statistically significant, *X*^2^ (1) = 0.33, *p* =.57, suggesting that the model including child SDH did not improve model fit. Therefore, results from the more parsimonious model are reported below (see Table 3).

**Table 3:** Results of the binary logistic regression predicting psychotherapy use

	B(SE)	95% CI for odds ratio		
		Lower	Odds ratio	Higher
Constant	−1.17 (0.32)***			
Medicaid Insurance^a^	−0.94 (0.18)***	0.27	0.39	0.56
Age	0.09 (0.02)***	1.04	1.09	1.14

Model *X*^2^ (2) = 43.68, *p* <.001. The present model was chosen over the model including SDH because the latter model did not improve model fit, and SDH did not significantly predict psychotherapy service use

^a^Reference group was commercial insurance

Seventy percent of the present sample did not access psychotherapy services, despite a co-occurring mental health condition implying their need. Child age increased the likelihood, *B* = 0.09, *z* = 3.52, *p* <.001; OR = 1.09; 95% CIs [1.04,1.14], while having public insurance decreased the likelihood of psychotherapy service use, *B* = −0.94, *z* = −5.22, *p* <.001; OR = 0.39; 95% CIs [0.27,0.55].

Descriptive analyses of psychotherapy claims are included in Table 4. Descriptive analyses of the present study’s sample who *did not* receive outpatient psychotherapy claims highlighted that most received applied behavior analysis (CPT code H2033, ABA code in Virginia in 2019), followed by office visits for evaluation of an established patient (at varying lengths of time; 99214, 99213), behavioral health day treatment (H2012), and mental health partial hospitalization (H0035).

**Table 4:** Characterization of psychotherapy services provided (*N* = 286 claims)

CPT Code	*N* (%)
90832 (one-to-one provision of therapy)	10 (3.50%)
90833 (one-to-one provision of therapy)	30 (10.49%)
90834 (one-to-one provision of therapy)	100 (34.97%)
90836 (one-to-one provision of therapy)	3 (1.05%)
90837 (one-to-one provision of therapy)	114 (39.86%)
90838 (one-to-one provision of therapy)	2 (0.70%)
90846 (family therapy)	5 (1.75%)
90847 (family therapy)	13 (4.55%)
90853 (group therapy)	9 (3.15%)
*Provider Specialty*	
Clinical psychologist	153 (53.50%)
Family practice	1 (0.35%)
Hospital	6 (2.10%)
Licensed Clinical Social Worker	49 (17.13%)
Neuropsychiatry	34 (11.89%)
Nurse practitioner	1 (0.35%)
Pediatric medicine	1 (0.35%)
Psych/Mental Health Facility	13 (4.55%)
Psychologist (billing independently)	19 (6.64%)
Unknown	9 (3.15%)
*Setting of Service*	
Telehealth	1 (0.35%)
School	3 (1.05%)
Office	254 (88.81%)
Home	1 (0.35%)
Group Home	4 (1.40%)
Off-Campus-Outpatient Hospital	1 (0.35%)
On-Campus Outpatient Hospital	3 (1.05%)
Community Mental Health Center	12 (4.20%)
Unknown/Missing	7 (1.00%)

### Model Diagnostic Assessment

Results of initial model diagnostic testing (i.e., inspection of studentized and standardized residuals) suggested strong model fit (e.g., only one case outside of acceptable range); however, model influence statistics suggested that commercially insured individuals exerted influence on the model. Importantly, most of the present sample were insured through Medicaid (72%). Given that these measures of influence were believed to represent true individual variability within these data, no datapoints were removed from analyses.

### Exploratory Post-Hoc Analyses

Post-hoc exploratory analyses were conducted to better understand these results. The present model findings were tested using other analytic approaches (i.e., chi-square and independent samples *t*-test) to assess the fragility of the model findings, given the high leverage points. Commercially insured individuals accessed psychotherapy services more frequently than those insured through Medicaid, _*X*_2 (1) = 31.18, *p* <.001. Additionally, older children (*M* = 11.91) were more likely to access services than younger children (*M* = 10.70), *t*(698) = −4.09, *p* <.001.

Medicaid insurance and child SDH were correlated with one another (*r* = -.37, *p* <.001). Results of an independent samples *t*-test indicate that child SDH differed between those having public insurance (*M* = 49.65) versus private insurance (*M* = 72.42), *t*(698) = 11.28, *p* <.001. To determine whether multicollinearity impeded the ability to detect significance, insurance was removed as a predictor from the model including SDH; however, model fit did not improve. Separate models to predict psychotherapy service use from substantive variables of interest were also conducted by insurance, and SDH did not significantly predict psychotherapy service use within either the Medicaid or commercially insured subsample.

To evaluate whether any of the individual domains of child SDH predicted the likelihood of accessing psychotherapy services rather than the overall score, individual regression analyses were conducted to evaluate whether each domain (education, health/environment, and social/economic) predicted psychotherapy service use when controlling for age and insurance type. No individual domain significantly predicted psychotherapy service use (*p*’s > 0.05).

## Discussion

To our knowledge, the present study is the first to systematically investigate the extent to which social determinants of health (SDH) contributed to psychotherapy treatment disparities among autistic youth with a co-occurring mental health condition. The overwhelming majority (70%) of the present sample did not receive psychotherapy services, despite having at least one co-occurring mental health condition. This suggests considerable unmet needs for psychotherapeutic treatment among autistic youth. Surprisingly, the results revealed that neighborhood SDH, as assessed by a validated, continuous measure of child-specific SDH, did not contribute to psychotherapy service disparities. Instead, age predicted the likelihood of service use, and public insurance was a barrier to psychotherapy service use.

For both autistic and non-autistic youth, best practice guidelines center psychotherapy in the treatment of most mental health conditions (Pettitt et al., 2022). However, in both groups, research suggests that rates of psychotherapy use are relatively low, especially when compared to rates of psychotropic medication management for mental health conditions (Rast et al., 2023; Stadnick et al., 2020; Young et al., 2019). The present findings indicated that most autistic youth with a co-occurring mental health condition were not accessing needed psychotherapy services. Identifying the similarities and differences between autistic and non-autistic youth’s service access may be important for understanding potential autism-specific barriers to care that were present within our sample.

In contrast to previous findings with non-autistic youth, neighborhood SDH did not contribute to psychotherapy treatment disparities among autistic youth in the present study. Research suggests that SDH, or the “nonmedical” factors that relate to health outcomes, contribute to mental health disparities in non-autistic samples, including predicting the likelihood of having a mental health condition (Ramachandran et al., 2023). In fact, research positions children’s neighborhood as a key contextual driver of child mental health conditions because of its effects on their exposure to violence, socioeconomic advantage, proximity to resources, and social connectedness (Alegría et al., 2022). A recent systematic review identified that specific neighborhood SDH also predicted the likelihood of using mental health services, including higher neighborhood SES, greater availability of health services, and urbanicity (Verhoog et al., 2022). For example, non-autistic youth living in communities where their system of care served mostly impoverished families were less likely to receive psychotherapy services than those who lived in communities with a more affluent-serving system of care (Fitts et al., 2019).

Conversely, in the current study of autistic youth, neighborhood SDH did not predict the likelihood of accessing psychotherapy services. Rather, rates of psychotherapy services were low for autistic youth in neighborhoods with low and high access to opportunity alike. These findings were surprising, given the trends in non-autistic samples as well as emerging data in autistic samples positioning neighborhood SES as a key predictor of service disparities among autistic youth (Drahota et al., 2020). One explanation for the present null finding may be that autistic youth universally have difficulty accessing services, as evidenced by the low rate of psychotherapy service use across neighborhoods. Prior research of autistic youth has revealed high rates of psychotropic medication use, particularly among those with co-occurring mental health conditions, such as ADHD (Coury et al., 2012; Jobski et al., 2017; Rast et al., 2023; Wiggins et al., 2021). Despite psychotherapy being the first line of treatment suggested for mental health conditions, autistic youth may access psychopharmacology more readily than other mental health treatment services (e.g., psychotherapy) because of the relative greater availability of community-based primary care providers (Jackel et al., 2017; Manter et al., 2025). Families may also find medication management to be more feasible than psychotherapy due to the lower frequency of appointments with providers.

In contrast, city- and national-level research highlights a scarcity of mental health facilities and providers serving autistic clients (Cantor et al., 2020, 2022; Cervantes et al., 2023; Maddox et al., 2021), and providers who work with autistic youth are often difficult to find and distant from families in need (Ning et al., 2019). Major contributors to the shortage of providers include their self-reported lack of knowledge and self-efficacy in working with autistic individuals (Corden et al., 2022), citing that they lack the “specialization” or “credentials” to work with autistic youth (Cervantes et al., 2023). Community mental health providers have noted the perceived ineffectiveness of available treatment strategies with autistic youth and the difficulties they have faced in coordinating care, which is often required when working with autistic youth due to co-occurring physical and mental health needs (Brookman-Frazee et al., 2012). The paucity of providers serving autistic youth is also echoed in caregiver and adults’ self-reports of their service access experiences (Camm-Crosbie et al., 2019; Chiri & Warfield, 2012). Considering this, neighborhood SDH may function differently for non-autistic youth, as non-autistic youth in higher resourced neighborhoods may be more likely to find providers who will accept them onto their caseload compared to non-autistic youth in lower resourced areas. However, autistic youth may only benefit from community-based mental health services that accept autistic clients onto their caseload.

While neighborhood SDH did not predict psychotherapy service use, insurance type was predictive of whether autistic youth accessed psychotherapy services. Specifically, youth with Medicaid insurance were less likely to access psychotherapy services compared to those with commercial insurance. These findings mirror those seen in the non-autistic population (Ali et al., 2019), as a limited number of mental health providers accept Medicaid, often due to the low reimbursement rates (Mark et al., 2020). Other reasons include the administrative burdens imposed by Medicaid compared to other insurances, including differences in allowable services, greater number of denials and subsequently greater paperwork (Dunn et al., 2024).

To further contextualize these findings, due to the present study’s use of Virginia claims data for the year 2019, approximately 30% of Virginia’s children were covered by Medicaid (Kaiser Family Foundation, n.d.). Additionally, Medicaid is the single largest payer of mental health services in the US (Centers for Medicare & Medicaid Services, n.d.). However, the most recent survey of psychologists from the American Psychological Association highlighted that only about 30% of providers accept Medicaid (American Psychological Association, 2022). Results from a multi-state study of Medicaid claims suggested that psychosocial services for children were highly concentrated to a small number of locations, which implied that individuals must travel to these specific treatment locations (Harati et al., 2020). Given that a key driver of service use is provider availability and accessibility, the scarcity of mental health providers accepting Medicaid directly contributes to service disparities. As autistic youth already experience difficulties finding providers who accept autistic clients, they may be especially vulnerable to the difficulties of finding a provider who also accepts Medicaid.

Another contributor to service disparities for both autistic and non-autistic youth is the critical lack of general child and adolescent mental health providers in the US. For instance, 59% of psychologists reported never working with children under the age of 11, and 37% reported never working with adolescents (13–17 years old) in a recent survey from the American Psychological Association (American Psychological Association, 2022). In the present sample, age predicted the likelihood of psychotherapy service use, which is likely in part due to the emergence of mental health challenges with maturation (Lai et al., 2019; Ryan et al., 2018; Turcotte et al., 2016). However, maturation cannot solely account for this finding because all participants in this study had a mental health condition, which would imply a need for services. Another reason that psychotherapy service use may increase with age for autistic youth with a co-occurring mental health condition may relate to clinical presentation. Previous research suggests that younger children are more likely to receive behavior management (e.g., applied behavior analysis), whereas adolescents are more likely to receive talk therapy (e.g., cognitive behavioral therapy; Ryan et al., 2018; Song et al., 2022). Given that younger children are more likely to exhibit behavioral signs of mental health conditions compared to adolescents, it may be that the combination of behavioral dysregulation and a diagnosis of autism inclines providers to refer children to services like applied behavior analysis rather than psychotherapy. However, future studies with data related to clinical presentation are needed to definitively understand differences in psychotherapy service use by age.

Considering that, apart from age, individual-level factors did not predict psychotherapy service use, the present findings suggest that we must look to the provider and systems-level to understand psychotherapy service use disparities for autistic youth. Factors like provider availability and accessibility impede the ability for both autistic and non-autistic youth, particularly those with Medicaid insurance, to access needed services. These barriers to care are further compounded for individuals on the spectrum who must pull from an even smaller pool of available providers. Therefore, the present findings underscore the need for policy reform to increase reimbursement rates and reduce administrative burden in order to incentivize more providers to accept Medicaid. The present findings also highlight the need for initiatives that bolster providers’ knowledge and comfort in working with autistic youth (e.g., Dreiling et al., 2022), as provider knowledge has been linked to an openness to accept autistic clients (Lipinski et al., 2022).

### Limitations and Future Directions

Though the use of insurance billing claims data has many strengths, the present findings are limited by the bounds of these data. Most notably, those included in the present study are likely only a subset of autistic individuals with a co-occurring mental health condition. Those who self-pay, use Tricare insurance, or did not have a claim associated with autism in the year of 2019 were not included in the present study due to the nature of single year billing claims data and how providers enter claims (i.e., typically using the diagnosis tethered to the individual service rather than a comprehensive list of all the child’s diagnoses). Sample characteristics related to diagnoses were also limited by the availability of diagnostic codes within the given year; thus, youth in the present sample may have other co-occurring conditions that were not accounted for in these data. Particularly, the low prevalence of autistic youth with intellectual disability within this sample was likely due to the dependence on the providers’ diagnostic billing codes to identify diagnoses paired with the present inclusionary criteria (i.e., both autism and mental health condition code within one year). Considering the prevalence of co-occurring intellectual disability among autistic youth in population-based samples (Maenner et al., 2023), it is likely that more youth in the present sample present with co-occurring intellectual disability than were identified using the available billing codes. Additionally, the age, insurance type, and zip code used in the present study were taken from the patient’s first claim of the year; however, these sociodemographic factors shifted for a nominal amount of individuals over the course of the year (0.29% reflect age maturation in their claims, 0.14% changed zip codes, 1.86% had multiple insurances).

Data related to race and ethnicity were not precisely reported, as 75% of the sample were missing ethnicity data and approximately 30% of the present sample were coded as having an “other” racial identity with no additional descriptor. Race categories were mutually exclusive; therefore, it may be that multiracial individuals were coded as having “other” race, but this cannot be confirmed. Additionally, many identities were underrepresented in the present data (e.g., American Indian/Alaska Native, Asian, or Native Hawaiian/Pacific Islander), leading to small cell sizes. Due to the aforementioned limitations, neither race nor ethnicity were included in the final models. Future studies with systematic data on race/ethnicity and greater representation across identities are warranted due to the known disparities in mental health service access by race and ethnicity (Broder-Fingert et al., 2013).

The use of the COI allowed for a multidimensional evaluation of SDH; however, not every dimension of SDH was captured within the COI. While the COI addresses many subdomains of SDH, data is lacking on neighborhood healthcare quality, prevalence of violent crime, civic participation, and neighborhood social capital and cohesion. The nature of secondary claims data analysis also precluded the collection of individuals’ report of SDH. Measures of individual SDH include key information regarding individuals’ daily experiences, such as food insecurity or transportation challenges (Billioux et al., 2017), that would complement the neighborhood-level SDH data included in the COI. Other individual-level factors that impact the likelihood of youth accessing psychotherapy services were also not available in these data, including the complexity of an individual’s profile or acuity of mental health conditions (Brookman-Frazee et al., 2018; Lecavalier et al., 2019; Salazar et al., 2015). Therefore, it may be that some individuals who were not receiving psychotherapy services were simply not good fits for an outpatient model of care at the time and may have benefited from inpatient services or stepwise models of care (i.e., to address behavioral acuity prior to mental health treatment). Additionally, individuals may have received psychotherapy services from sources unaccounted for in these analyses, such as public early intervention services or school-based supports that were not billed through insurance. These data were also cross-sectional and do not account for variation in service use patterns over time.

Finally, the present regression models included datapoints with high influence, which were determined to be largely associated with commercial insurance. High influential datapoints can impact the robustness of regression model findings; however, the primary findings related to differences in service use by insurance type and age were replicated using different analytic approaches. While replication in other samples is warranted, finding the same pattern of results across analytic approaches supports the strength of these findings.

Future studies would benefit from multiple years of claims data to address some of the aforementioned limitations imposed by the present data. Multiple years of claims data are preferable for the identification of individuals with clinical diagnoses of interest, such as autism (Chronic Conditions Warehouse, 2024). Multi-year studies will be better positioned to use more robust measures of service use, such as volume of mental health services across time or continuity of mental healthcare, rather than the dichotomous measure of service use used in the present study.

The present data comprised services claims from the year prior to the COVID-19 pandemic, which abruptly changed mental health service delivery. The rise in telehealth services may have increased the feasibility and accessibility of mental health services for children in lower resourced areas by mitigating the burden of neighborhood availability of providers, available transportation, and time to participate. However, considering the variability in families’ responses to telehealth service delivery (White et al., 2021), future studies investigating telehealth services’ relationship to neighborhood resources and mental health service use are needed within autistic samples.

Future studies with more specific measurement of service use are warranted to better understand the present results. Specifically, the majority of autistic youth in the present sample did not access psychotherapy services, and prior research highlights the high rates of psychotropic medication use within samples of autistic youth with a co-occurring mental health condition (Jobski et al., 2017). Future research would benefit from the collection of specific datapoints regarding the type of services used (e.g., type of psychotherapy: parent behavior management training versus individual talk therapy), type of medication (e.g., antidepressant versus stimulant), and clinical outcomes (e.g., chronicity of mental health challenges, emergency department visits)—such as may be the case in studies using electronic health records. These data would allow for the specific evaluation of what mental health treatment services autistic youth access (i.e., first or second line treatment), whether treatment decisions differ by SDH, and how treatment decisions influence health outcomes across varying SDH. Given that the null finding of SDH and mental health service use diverges from those in non-autistic samples, comparative studies are also warranted to determine if neighborhood SDH functions differently between autistic and non-autistic youth with a mental health condition.

## Conclusions

The present findings indicated that while neighborhood SDH did not predict the likelihood of autistic youth accessing psychotherapy services, having public insurance related to a decreased likelihood of accessing needed psychotherapy services. In addition, age was associated with an increased likelihood of accessing these services, which may be due to an inclination towards behavior analytic approaches for autistic children compared to adolescents. Autistic and non-autistic youth insured through Medicaid share the challenges related to limited provider availability. However, autistic youth experience the additional barrier imposed by the shortage of providers accepting autistic clients, which simply exacerbates preexisting barriers to mental healthcare. The present findings underscore that the likelihood of autistic youth with a co-occurring mental health condition receiving needed psychotherapy services rests largely in the hands of the system rather than the individual. Future research and practice initiatives must prioritize advocacy for insurance reform to incentivize providers to accept Medicaid and a bolstering of providers’ knowledge and comfort in working with autistic youth.

## Electronic Supplementary Material

Below is the link to the electronic supplementary material.

Supplementary Material 1

## Footnotes

1 Prior autism research underscores the importance of asking study participants their preferred terminology; however, given that the present research study did not allow for discussion with participants, we have followed the recommendations outlined in the literature regarding autism terminology (Botha et al., 2023; Bottema-Beutel et al., 2021).
