# Supplementary material for: Sociodemographic Factors Associated with Autistic Youth’s Psychotherapy Service Use
Source: Adm Policy Ment Health. 2025 Jun 3;52(4):714–26. doi: 10.1007/s10488-025-01448-9 (PMC12310900; doi:10.1007/s10488-025-01448-9)
Supplement: Supplementary file 1 — Supplementary Material 1 [file 10488_2025_1448_MOESM1_ESM.docx]

**Supplemental Materials A**

*Childhood Opportunity Index 2.0 Indicators and Descriptions by Domain (Education, Health/Environment, Social/Economic)*

| **Indicator** | **Description** |
| --- | --- |
| *Education* | |
| ECE Centers | The number of ECE centers within a 5-mile radius. |
| High-quality ECE centers | The number of accredited ECE centers within a 5-mile radius. |
| ECE enrollment | Percent 3- and 4-year-olds enrolled in ECEs. |
| Third grade reading proficiency | The percentage of third graders earning proficient scores on standardized reading assessments. |
| Third grade math proficiency | The percentage of third graders earning proficient scores on standardized math assessments. |
| High school graduation rate | The percentage of ninth graders graduating from high school on time. |
| Advanced Placement Enrollment | Ratio of students enrolled in at least one AP course to the number of 11^th^ and 12^th^ grade students. |
| College enrollment in nearby institutions | Percentage of college-aged students enrolled in college within a 25-mile radius. |
| School poverty | Percentage of students eligible for free or reduced-price lunches (reversed). |
| Teacher experience | Percentage of teachers within their first or second year (reversed). |
| Adult education attainment | Percentage of adults aged 25 or older with a college degree or higher. |
| *Health and Environment* | |
| Access to healthy foods | Percentage of households without a car located further than a half-mile from the nearest supermarket (reversed). |
| Access to green space | Percentage of impenetrable surface areas (e.g., rooftops, roads, parking lots; reversed). |
| Walkability | EPA Walkability Index |
| Housing vacancy rate | Percentage of vacant housing units (reversed). |
| *Social and Economic* | |
| Poverty rate | Percentage of individuals whose household income is below the federal poverty threshold (reversed). |
| Public assistance rate | Percentage of households receiving cash public assistance or Food Stamps (reversed). |
| Homeownership rate | Percentage of owner-occupied housing units. |
| High-skill employment rate | Percentage of individuals aged 16 or older who are over employed in high-skill occupations such as management, business, finance, science, education, engineering, community service, and more. |
| Median household income | Median household income. |
| Single-headed households | Percentage of family households that are single-parent headed (reversed). |

*Note.* This table was abstracted from the Childhood Opportunity Index technical document and modified for simplicity (Noelke et al., 2020)
